# The London County Council and the Tottenham Hospital

**Published:** 1902-11-15

**Authors:** 


### The Hospital.

A Journal of

The Medical Sciences and Hospital Administration.

Vol. XXXIII.—No. 842.] Edited by Sir Henry Burdett, K.C.B., and Solomon C. Smith, M.D., M.R.C.P. [November 15, 1902.

### THE LONDON COUNTY COUNCIL AND THE TOTTENHAM HOSPITAL.

It appears from a statement in the annual report of the Tottenham Hospital that the migrations of the population of North and North-east London into Tottenham have of recent years converted what were formerly pleasant rural districts into an immense residential neighbourhood, without any additional provision for hospital accommodation. The population of the district served by the Tottenham Hospital, the only institution of its kind within this area, already numbers 300,000. Very soon, however, the number of residents will be considerably augmented by the action of the London County Council, which proposes to transfer to suitable dwellings, specially erected for their accommodation in the Tottenham district, over 40,000 people from the congested districts of London. The Tottenham Hospital contains at the present time about eighty beds, and on a most moderate computation, the existing population should have available for the accommodation of the sick, in a well-equipped general hospital, 300 beds, or nearly four times that number. Every effort is being made, with encouraging success, by the authorities of the Tottenham Hospital to make their bed accommodation adequate to the needs of the people whom the hospital serves. But, so far, no communication has been received from the London County Council, who clearly ought to recognise the duty imposed upon them by their action in moving upwards of 40,000 new people into the Tottenham district, to make adequate provision, by the erection of buildings in connection with the Tottenham Hospital, for a proper supply of hospital beds to take the more urgent cases of sickness which must necessarily arise in a large population, for the welfare of which the London County Council must be held responsible.

This journal was brought into existence to maintain the voluntary system of hospital support, and we would never consent to support any proposal which was calculated to interfere with the maintenance of the great general hospitals by voluntary contributions. The conditions, however, under which we live are constantly changing, and the problem of providing adequately for the healthy residence of masses of the population in a great city like London entails obligations upon the municipality which must necessitate material changes before an adequate settlement can be secured. One change is, in our judgment, that the London County Council, when it undertakes the responsibility of moving tens of thousands of persons of both sexes and all ages from a congested district into the suburbs, must recognise that it not only has to secure for the new comers suitable houses, replete with the best water, heating and lighting arrangements, but at the same time to take care that adequate provision in cases of sickness is also forthcoming in the centre selected for the new population for which they thus become responsible.

At Tottenham during the last three years the Hospital Committee have been successfully working to provide through the free-will offerings of the people an adequate revenue to maintain the additional beds which they have opened, and which now amount to 80—*i.e*, three times the number available when they took over the hospital. There is every prospect, from the experience the Committee have gained, that the population will provide the requisite revenue for the maintenance of an adequate number of hospitals beds, providing funds are forthcoming to defray the initial cost of the necessary buildings on the excellent site which the Committee have under their control. This circumstance renders it possible for the County Council to step into the breach, to enter into an agreement with the Tottenham Hospital to erect and equip the necessary buildings to meet the requirements of the new population they are introducing into the district, and to provide that the Committee will undertake to maintain these additional beds when opened, with the assistance of the contributions of the population for whose benefit they have been supplied. Such a step would be welcome, because it would show that the London County Council is alive to the responsible duties which devolve upon its members, and that they are prepared to wisely meet the claims of the poorer citizens for whom they have provided model dwellings. We shall watch with genuine interest the development of this new aspect of the hospital question at Tottenham, because it may initiate changes which must tend to consolidate and uphold the voluntary system, whilst they wisely save the ratepayers from annual charges, which it is desirable in the interests of all classes of the community shall be met by voluntary subscriptions.

